# Factors influencing physical healthcare quality for people with intellectual disabilities: psychiatry multidisciplinary team perspective

**DOI:** 10.1192/bjo.2025.10051

**Published:** 2025-06-30

**Authors:** Madiha Majid, Stefan Rennick-Egglestone, Bronwyn Harris, Ashok Roy, Hayley Crawford

**Affiliations:** School of Health Sciences, Institute of Mental Health, University of Nottingham, UK; Warwick Medical School, University of Warwick, UK; Coventry and Warwickshire Partnership NHS Trust, Coventry, UK; NIHR Nottingham Biomedical Research Centre, Institute of Mental Health, Nottingham, UK

**Keywords:** Intellectual disability, learning disability, psychiatry of intellectual disability, multidisciplinary team, qualitative research

## Abstract

**Background:**

There is a need for better collaborative care between services to improve healthcare provision for people with intellectual disabilities. In the UK, the learning disability psychiatry multidisciplinary team (MDT) is a specialist team responsible for providing and coordinating care for people with intellectual disabilities.

**Aims:**

To document learning disability MDT perspectives on factors influencing healthcare quality for people with intellectual disabilities.

**Method:**

Healthcare professionals who were members of a learning disability MDT within a National Health Service Trust in the West Midlands were purposively sampled for interview (*n* = 11). Participants included psychiatrists, nurses, occupational therapists and speech and language therapists. Data were analysed thematically using Braun and Clarke’s six-stage approach.

**Results:**

Factors influencing the quality of healthcare provision included: the learning disability MDT working to overcome systemic barriers; the consequences of specific failures within mainstream healthcare services, such as diagnostic overshadowing; inadequate use of information collated in health passports; and inadequate capacity assessments of people with intellectual disabilities. Improvements in healthcare provision for people with intellectual disabilities require better accessibility to healthcare and better training for healthcare professionals so they can understand the health needs of people with intellectual disabilities.

**Conclusions:**

A rapid review of practices around health passports for people with intellectual disabilities should be conducted. Healthcare professionals working in mainstream healthcare services need an increased awareness of the harms of diagnostic overshadowing and inadequate capacity assessments. Conclusions are based on findings from MDTs within one health board; future work may focus on understanding perspectives from different teams.

People with intellectual disabilities face several health inequalities, illustrated by a higher level of multimorbidity and significantly reduced life expectancy when compared with the general population.^1–5^ There are a range of explanations for health inequality, which include health needs that differ from those of the general population, vulnerability when care needs are considered and services that do not adequately meet the needs of individuals.^1,6^ People with intellectual disabilities are more likely to experience barriers, such as communication difficulties when accessing healthcare services,^7^ with reasonable adjustments not being made.^8^ The COVID-19 pandemic illustrated the consequences of health inequalities, patient vulnerability and barriers to accessing effective care, because those with intellectual disabilities were more likely to be admitted to hospital and eight times more likely to die from COVID-19 than the general population.^9^

## Barriers to care influence the quality of healthcare provision

Qualitative studies in the UK have identified that barriers to care are widespread and exist in different healthcare settings. Barriers in acute care, primary care and secondary care for people with intellectual disabilities have included experiencing stigma from staff, distress from prolonged waiting times and exclusion from decision-m(aking.^10,11^ The barriers and discrimination that patients experience extend to clinical consultations, where their presentation is often misattributed to the intellectual disability diagnosis.^12^ Clinician factors have been cited as significant barriers,^13^ which include a lack of training on, and knowledge of, the nature of intellectual disabilities,^10,11^ amplifying communication difficulties, and are thus indicative of serious training gaps in managing physical health complaints in people with intellectual disabilities.

Barriers to care are also experienced by those caring for people with intellectual disabilities (including formal and informal carers), who are often involved when the need arises for accessing healthcare.^14^ These include navigating the complexity of the healthcare system in the UK, the referral processes and information about services.^12^ Carers can feel excluded from clinical decisions, with their knowledge of the patient and lived experience often disregarded.^15^

These barriers in the health sector are underpinned by the need for better collaborative relationships between those involved in the care of people with intellectual disabilities.^10^ It is therefore crucial that we better understand the experiences of those working in services that provide care for people with intellectual disabilities.

## The learning disability multidisciplinary team

The psychiatry of intellectual disability multidisciplinary team (MDT), which is commonly referred to as the learning disability team in the UK, is typically composed of psychiatry doctors, psychologists, occupational therapists, nurses, healthcare assistants, pharmacists and speech and language therapists.^16^ In addition to supporting the mental health needs of those with an intellectual disability, the MDT is well placed to develop understanding of specific service user needs and facilitate engagement with other parts of the healthcare system.

Members of the learning disability team are directly involved with coordinating physical healthcare and liaising with different healthcare professionals as part of their specialist role. There is limited research on learning disability MDT perspectives on care provision for people with intellectual disabilities. Their unique position and role may add further insights into identifying barriers and enablers for patients when accessing physical healthcare. Exploring this may support existing findings or help identify new themes to underpin improved care. Furthermore, identifying and evaluating the standard of the service, and how the learning disability MDT work collaboratively with other healthcare providers, is important to understand, so that progress in improving patient care can be made.

## Aims and objectives

The aim was to document learning disability MDT perspectives on factors influencing quality of healthcare for patients under their care. The objectives were to:

- understand the role of the learning disability MDT in facilitating physical healthcare;
- identify enablers and barriers to effective healthcare;
- identify areas of improvement both within the learning disability MDT and the healthcare system in which it operates.

## Method

### Ethical considerations

The authors assert that all procedures contributing to this work comply with the ethical standards of the relevant national and institutional committees on human experimentation, and with the Helsinki Declaration of 1975 as revised in 2013. All procedures involving human subjects/patients were approved by the University Biomedical and Scientific Research Ethics Committee criteria at the University of Warwick for course-delegated ethical approval (no. REGO2021_MPH_002).

Written informed consent was obtained from participants.

Where specific patient safety concerns were mentioned by clinical staff, the lead author verified that these had been escalated through the appropriate channels.

### Setting

Interviews were conducted within the learning disability service in Coventry and Warwickshire Partnership NHS Trust in England, UK.

### Participants

Staff from the learning disability teams included both in-patient and out-patient (community) MDTs (*n* = 12 teams) within the service. Staff that did not regularly work with the team, such as bank staff, were not included. Some staff members were part of more than one learning disability MDT within the trust.

Purposive sampling was utilised in this study.^17^ The aim was to encompass a heterogenous sample to include diversity in role, and reflect the various specialists involved in the MDT. An email invitation was circulated by MDT administrators. The interview sample consisted of 11 participants, to encompass most MDT roles, and included 2 psychiatry doctors, 4 in-patient and out-patient learning disability-trained nurses, 1 healthcare assistant, 2 occupational therapists and 2 speech and language therapists.

### Data collection

Semistructured interviews, based on the interview guide shown in Table 1, were conducted either virtually or in a private clinical setting by the primary researcher, a female psychiatry trainee who has in-depth awareness of the learning disability MDT and its functioning; this allowed for wide-ranging and relevant questions for the MDT narrative to be explored. Data were collected from February to April 2022.

**Table 1:** Interview guide

Introduction and brief overview	Establish role: what is your role and how long have you worked in this role? How long have you worked for the current team?
Key questions	How does your role facilitate healthcare for patients with learning disabilities? What has been your experience of collaboration with other disciplines? In your experience, have you felt there have been any barriers to ensuring that service users receive optimum physical healthcare? If so, why do you think this is the case? Based on your experiences, what is working well within services to reduce barriers to physical healthcare? Based on your experiences, how do you feel that services can be developed to improve physical healthcare for service users? How has COVID-19 impacted barriers to physical healthcare for service users?
Conclusion	Summarise discussion. Is there anything important that you have not mentioned?

Interviews, ranging from 15 to 30 min in length, were recorded, transcribed and pseudonymised. Participant quotes have not been identified by role, to avoid identification.

### Data analysis

Using a six-phase framework,^18^ a thematic analysis was conducted. An inductive approach was adopted, given the scarcity of research into care for people with intellectual disabilities as perceived by learning disability MDTs; from the data, this approach allowed for an understanding of the MDT functioning and perspective, how it interacts and the challenges faced when navigating care for people with intellectual disabilities.

Two researchers (M.M. and B.H.) developed initial codes; these were agreed and consensus reached through open discussion. B.H., a qualitative researcher, verified that codes were comprehensible to those external to mental health services. Where there were disagreements, these were discussed with the third researcher (H.C.). Although coding was mostly semantic, when participants appeared to express emotions when discussing a topic a latent approach was used.

As we selected themes, we privileged identification of concepts that would be recognisable across healthcare services. The primary researcher’s (M.M.) clinical experience of learning disability MDTs enabled prioritisation of theme selection. M.M. developed recommendations from the data and through discussion with the authors.

## Results

We identified five themes: the learning disability MDT working to overcoming systemic barriers, communication with patients, the role of mainstream healthcare services, capacity to make health decisions and improving access to healthcare (detailed in Table 2).

**Table 2:** Themes and subthemes

The learning disability multidisciplinary team working to overcome systemic barriers	Operating in a healthcare system where health inequalities exist Facilitating healthcare Multidisciplinary team values and processes
Communication with patients	Patient factors Hospital passports Impact of COVID-19
The role of mainstream healthcare services	Emergency and secondary care Primary care services Organisational culture and attitudes
Capacity to make health decisions	Assessing capacity Best-interest decisions
Improving access to healthcare	Accessibility of healthcare Health promotion and disease prevention Training for healthcare professionals

### Theme 1: the learning disability MDT working to overcome systemic barriers

#### Operating in a healthcare system where health inequalities exist

MDT members were strongly aware of regular difficulties faced by patients with intellectual disabilities in accessing healthcare services. The quality of care provided to patients was also seen as poor where they were able to access services. Having an intellectual disability was described as a barrier per se in accessing care, frequently compounded by individuals having additional diagnoses such as autism or attention-deficit hyperactivity disorder (ADHD):

‘One of the biggest challenges for the people that we work with is that their underlying physical health needs aren’t investigated sufficiently’ (participant F).

One participant cited that accessibility to healthcare had improved over recent years. Most participants expressed frustration about significant issues in healthcare settings:

‘Our patients don’t just have learning disabilities, they usually have other problems like autism, ADHD and other things which make it much harder for them to just sit and access primary care like you and me’ (participant D).

Participants described clinical scenarios in which patients were insufficiently investigated or treated in mainstream health services (healthcare services external to the specialised care provided by the learning disability MDT).

#### Facilitating healthcare

MDT members viewed themselves as advocates for physical healthcare, and there was a shared understanding that each team member’s expertise was fundamental in providing good-quality patient-centred and holistic care.

Within in-patient settings where the person was non-verbal, in-depth understanding was vital to recognising when the physical health of that individual was deteriorating:

‘I think I am quite good and skilled at knowing when our patients are acting differently, pinpointing that it is not only a mental health issue or behavioural, but there could be something underlying physically’ (participant E).

When liaising with mainstream healthcare services, staff felt that their knowledge of the person and their recommendations were not always taken into consideration, which led them to feeling both stigmatised and their expertise ignored, particularly where significant team resources were required in making recommendations.

#### MDT values and processes

Team members described an open and supportive working environment where they felt able to express their viewpoints with each other. Working well together was seen as vital in ensuring they achieved their shared goal of providing good patient care. MDT meetings were opportunities to discuss physical health concerns of patients. The structure of specialist services provided by the team, such as the ‘Behaviour Clinic’, sought to specifically identify physical healthcare needs as part of the assessment of the patient, given the inextricable link between physical health and mental health:

‘We have a really good process with nursing around the behaviour clinic in really making sure that we’re focusing on, have really explored what the health needs are, the physical health needs first of all’ (participant F).

The MDT viewed itself as crucial in integrating care and maintaining collaboration between different services such as primary care and social care.

Liaising with different healthcare providers and maintaining positive relationships with them were enablers to care, particularly important where the person’s condition was complex or they had additional care needs:

‘It’s always easier when you start to know nurses in other teams or doctors and other teams by their first name you can have a conversation with them and you, can so I think be more amenable or open’ (participant I).

There were challenges in coordinating care between different specialists, particularly where collaborative working between multiple professionals was required:

‘You don’t always know who to contact really. So that’s a barrier in itself I think’ (participant E).

Working in a health service with limited resources had an impact on the team’s workload and capacity, particularly at times when they were receiving many referrals. Staff also mentioned blurring of roles and responsibilities when they had to take on or support the role of others that would not necessarily fall within their remit.

Referrals to the MDT deemed ‘inappropriate’ were mostly attributed to insufficient physical health investigations being conducted prior to referral.

### Theme 2: communication with patients

#### Patient factors

Having an intellectual disability may mean difficulties in explaining symptoms, such as expressing pain and describing the nature of it. Literacy difficulties may cause anxieties in a hospital setting:

‘People don’t have the tools to be able to explain what their pain feels like, where it’s located, whether it’s a dull ache or a sharp pain’ (participant J).

Additionally, communicating with healthcare professionals or being in a healthcare setting may cause distress to individuals. These factors were seen as impacting whether examinations were conducted thoroughly or conducted at all, and whether people were able to tolerate or comply with investigations and treatment. Delays in diagnosing and managing physical health concerns were commonly mentioned.

Furthermore, the power dynamic between clinicians and people with intellectual disabilities was made evident due to team members or carers having to advocate for individuals to receive investigations or treatment, rather than people with intellectual disabilities being empowered to access and utilise services as readily as those without intellectual disability.

#### Hospital passports

A hospital passport is a document detailing the patient’s health needs, including useful information such as communication and reasonable adjustments that may be needed, for example, in a healthcare setting.

Hospital passports were sometimes overlooked in emergency or secondary care settings, with potential for serious consequences. MDT members were perplexed that this was happening given the time and effort that went into producing these documents, which would be helpful for both the clinician and the patient in providing a good standard of care:

‘We’ve had people going to hospital who have dysphagia, that are on a pureed, or a mixed or moist diet for example and have been given a sandwich. All you had to do is look at their hospital passport and see that what diet they’re on, like you could have killed them’ (participant H).

There was an assumption that an individual with intellectual disabilities who was able to engage in conversation did not have communication difficulties and therefore adjustments in communication were not deemed necessary, despite this being detailed on their health passport. Many felt that this indicated a lack of knowledge or training when assessing people with intellectual disabilities, because it was treated as a hidden disability. Again, this was seen as contributing to a delay in diagnosis and treatment:

‘I’ve seen some really, very experienced doctors and nurses talking at great length about things and trying to explain and reason with people with learning disabilities when really, they’re not capable of understanding that level of speech. And really, they should know better than that’ (participant J).

#### Impact of COVID-19

During the COVID-19 pandemic, the teams had to adjust to remote consultations. This change occurred in the context of limited internet access, poor facilities available to enable remote use and digital exclusion for patients:

‘A lot of people with disabilities are socially deprived, they don’t have access to the stuff that they need to, they don’t have a laptop, computer or even a decent phone to be able to get on a video call either’ (participant H).

The use of face masks by clinicians hindered communication:

‘and a lot of people need that total communication environment when they can’t see your facial expression, and your voice is maybe a little bit muffled, they don’t get some of the really crucial information for them to be able to understand’ (participant J).

There was a benefit for the team, because remote meetings meant that many professionals were able to simultaneously attend meetings and therefore coordinate care more efficiently.

When discussing the impact of COVID-19, the levels of intellectual disabilities and co-occurring diagnoses, such as autism or ADHD, were described but not specifically mentioned. This indicated that experiences were not central to their diagnostic labels, or that disentangling presentations and attributing it to an intellectual disability or autism diagnosis may be difficult.

### Theme 3: mainstream healthcare services

#### Emergency and secondary care

Routine visits to a hospital, when planned in collaboration with secondary care teams, were often positive experiences. For emergency service visits, care experiences were often variable; long waiting hours were described as inappropriate when a patient was anxious or displaying behaviours that challenge:

‘There is a delay in treatment, proper delay in treatment when they go to hospital, because of challenging behaviours’ (participant B).

In some instances, this was despite staff contacting the department prior to attendance requesting reasonable adjustments for the patient, and despite relaying the same information to hospital staff by those accompanying the patient. Reasonable adjustments were, however, noted to be made after behaviours that challenge had been displayed – for example, after verbal aggression was seen in the waiting room. It appeared that only when others in the emergency service waiting room were experiencing discomfort that the distress of the person with an intellectual disability was acknowledged and then seen as requiring reasonable adjustments.

#### Primary care services

There were positive experiences highlighted in primary care, such as with GPs carrying out annual health checks and actively seeking communication needs from the MDT or individual prior to appointments. However, not all primary services made reasonable adjustments. Additionally, MDT requests to primary care for patient reviews, investigations or implementation of medication changes were either not actioned or there were significant delays in doing so:

‘GPs flat out refused to come and see people at home, those people who desperately need home visits that can’t get to the GP for whatever reason, because of their physical health or their sensory needs, or it changes their routine, or it is too upsetting, it could be any manner of things but that’s a reasonable adjustment to go and see them at home’ (participant H).

#### Organisational culture and attitudes

The negative attitudes faced by people with intellectual disabilities from healthcare professionals in mainstream healthcare services was also felt by staff who accompanied individuals in these settings. The impact of these attitudes was seen as contributing to delays in investigation and treatment:

‘We’ve had doctors say to our team oh they’re not normal, are they? That’s a really ingrained thought process that is hard to change’ (participant C).

There were several examples of diagnostic overshadowing,^19^ where the underlying cause of the presentation of an illness was incorrectly misattributed to the patient’s diagnosis of intellectual disabilities, and this meant insufficient or delayed investigations:

‘When I mention it, they look at me as if I’ve gone crazy, and I kind of just, “she could be menopausal, think about the hormonal changes”. Nobody has even considered that women with a learning disability would experience the menopause, who’d have thought it?’ (participant F).

### Theme 4: capacity to make health decisions

#### Assessing capacity

MDT members may assist clinicians external to the team with completion of capacity assessments. Capacity assessments in mainstream health services were not always conducted adequately, or the incorrect process was followed, indicating poor understanding of the Mental Capacity Act 2005 (MCA).^20^ Some medical decisions were made without assessing capacity to consent to treatment or investigations. The outcome of an inadequate assessment may have resulted in a decision not to pursue important investigations or treatment:

‘They were making it difficult to discharge her, they were asking us if she has capacity, she doesn’t have the capacity, but they wanted us to sign her off, or her to sign the self-discharge, but it does take time to assess capacity, but they don’t take that time’ (participant C).

There was difficulty in ascertaining what people were thinking when they were unable to express them. A person with an intellectual disability may have chosen to make a ‘bad decision’ relating to their physical health that called into question their capacity if the decision was not in keeping with the assessor’s values.

The crux of these difficulties in assessing capacity follows on from issues with communication discussed in theme 2, such as failure to present information in an inappropriate format and adequately assess understanding. Difficulties were also thought to stem from negative attitudes of external healthcare professionals and lack of skills and resources, further linking in with previous themes:

‘If you gave it to them in a different format, they might very well then be able to make an informed choice about their care’ (participant H).

‘Individual teams could have access to a library of pictures which allowed them to show what some of the procedures would mean … or what a piece of equipment would look like. You know, to be able to hold it or see it’ (participant I).

#### Best-interest decisions

A ‘best-interest meeting’ occurs when an adult lacks the capacity to make a decision themselves and would need others to make it on their behalf.^20^

MDT members felt that the responsibility for organising these meetings and the decision-making process did not lie with the learning disability team, although they were also of the opinion that this expectation was there from external healthcare services. This was in the context of limited resources, where it was felt that resources within the team were stretched:

‘People want us to do the capacity assessment for them and to make all the decisions for them and do all the paperwork for them, even though it’s clearly their decision’ (participant J).

‘It’s a very common experience for us, that GPs can be very reluctant to make a best interest decision, I suspect it’s because they don’t fully understand capacity regulation … We’re not the decision maker when it comes to physical health stuff’ (participant F).

This reflected a lack of training and resources within mainstream healthcare settings, and therefore the reluctance to prioritise and take responsibility for organising and conducting best-interest meetings to inform a clinical decision.

### Theme 5: improving access to healthcare

#### Accessibility of healthcare

The need for healthcare services to make reasonable adjustments when requested, and to do so consistently given the obstacles encountered in these environments, was a common improvement that was suggested:

‘then they need more time in the consultation and less time in waiting because waiting is usually in a noisy and difficult environment, and it really causes anxiety’ (participant D).

Liaison nurses were seen to be a valuable resource in supporting consultations in hospital settings, and as an important collaborative link between the learning disability MDT and the medical team at the hospital. They were also described as enabling those who access services to do so more effectively.

#### Health promotion and disease prevention

Preventative healthcare measures were overlooked, with screening services not being prioritised. Participants described that not enough was being done by healthcare services to engage people with intellectual disabilities in preventative services. While many supported the annual health check in primary care for monitoring health and identifying health issues, it was felt by others that this was insufficient to meet the health needs of people with intellectual disabilities.

Poor lifestyle choices and the physical health comorbidities of individuals were attributed to a poor understanding of health behaviours and their consequences. This was related to health promotion and disease prevention for people with intellectual disabilities being perceived as inadequate:

‘I think we need to do more really in terms of education and health promotion for the patients’ (participant E).

#### Training for healthcare professionals

A positive interaction from staff in mainstream healthcare services depended on staff understanding of intellectual disabilities or previous experience of working with people with intellectual disabilities. Interactions with mainstream healthcare services indicated poor healthcare professional training, which meant that care was inconsistent across different healthcare settings and reasonable adjustments were not always made.

Participants discussed the idea that training may help healthcare workers approach patients with compassion and positive attitudes, allowing for more focused consultations.

## Discussion

### Principal findings

The learning disability MDT is important in bridging the gap between services in regard to advocating for physical health and coordinating between services. A wide range of barriers and enablers were identified from the learning disability MDT perspective. Significant barriers included poor accessibility to healthcare, mainly in acute services and primary care, because reasonable adjustments were not always made, despite being required by the UK Equality Act 2010.^21^

Many described the negative attitudes faced by people with intellectual disabilities in various mainstream healthcare settings, and attributed this to both the culture within mainstream services towards people with intellectual disabilities and professionals’ lack of training and understanding of intellectual disabilities. Processes for assessing capacity to consent to investigations or treatments in these settings was discussed as being frequently inadequate. Communication with individuals was a significant barrier, despite the availability of care documents such as health passports. The impact of these barriers often resulted in diagnostic overshadowing and suboptimal care. Barriers were discussed in the context of poor health promotion and disease prevention in this population. There were examples of positive experiences, particularly when learning disability liaison nurses were involved and able to support consultations.

### Areas where improvements are required

Many of the findings are in line with previous qualitative research.^12,22,23^ From our findings and published literature, we recommend the following steps.

### Healthcare should be made more accessible

**Recommendation 1: systemic failures around healthcare passports should be investigated and rectified.** Health passports, when used, are vital documents that provide valuable information about individuals^24^ to enhance care and improve their safety; MDT members felt that these should function as a key document in health services. There is a need to standardise health passports for people with intellectual disabilities, given the variation in information included on these passports.^25^ The development and standardisation of health passports with the learning disability MDT also presents an opportunity for different services who are involved in the care of the patient, such as primary care, to collaborate and improve access to healthcare.

**Recommendation 2: develop and utilise the learning disability liaison nurse role within mainstream hospitals.** Development of liaison nursing care to reduce barriers to care has been highlighted as an area requiring improvement.^26^ Involvement of a learning disability liaison nurse in the care of patients in general hospitals is more likely to result in reasonable adjustments being made.^27^ Utilising the liaison nurse role has been shown to be both valuable and achievable,^28^ and therefore may be a central role through which learning disability MDT members can liaise, in addition to being a direct source for healthcare professionals from other healthcare settings to coordinate care.

### Mainstream healthcare professionals need focused training

**Recommendation 3: improve support and training for assessing capacity for healthcare professionals in mainstream healthcare settings working with people with intellectual disabilities**. Capacity assessments in relation to the inadequate completion of investigation and treatment in mainstream services is of great concern. There are many obstacles, such as person-specific and systemic barriers, involved in the complexity of assessing capacity in those with intellectual disabilities.^29,30^ Support in the form of practical applications should be developed for professionals in mainstream services involved in assessing capacity.^29,31^

**Recommendation 4: increase awareness of the harms of diagnostic overshadowing.** Examples of clinical presentations being attributed to intellectual disabilities diagnosis, with valid concerns from both individuals and staff being overlooked, indicated a lack of awareness of the health needs of people with intellectual disabilities. Oliver McGowan Mandatory Learning Disability and Autism Training has been implemented for healthcare staff in England.^32^ Clinicians could, however, specifically benefit from formal training in the health needs of people with intellectual disabilities, with an increased understanding of the consequences of diagnostic overshadowing; this will encourage them to provide systemic assessments, enabling safe and effective care.

### Thinking beyond the annual health check

**Recommendation 5: annual health checks should complement wider public health initiatives.** Participants pointed out that the annual health checks offered to people with intellectual disabilities carried out in primary care are important, yet insufficient to promote healthy lifestyles. Annual health checks, while vital for identifying unmet health needs and improving access to healthcare, are unlikely to lead to follow-up or provide adequate support and advice for those advised to change their lifestyle, or for those expressing difficulties in making or sustaining healthy lifestyle changes.^33^ This may represent an opportunity for increased support for help primary care clinicians to promote good health with the resources to provide follow-up.

**Recommendation 6: health promotion and disease prevention initiatives should be adapted to, and focused on, the needs of people with intellectual disabilities.** This may involve tailoring initiatives to meet communication needs, providing reasonable adjustments and engaging carers or family.^34^ Participants spoke about poor health being multifactorial, but specifically that poor understanding of health behaviours led to poor health outcomes, relating this to inadequate public health initiatives. Action should also focus on addressing the social determinants of health from an early age, along the life-course, to improve health outcomes, which can be influenced by upstream changes.^35^ Much work is needed on downstream changes in health promotion: intellectual disabilities population-specific intervention studies are few and far between,^36,37^ and may present a missed opportunity to involve learning disability MDT specialists in research and public health interventions.

### Collaborative care between services needs to be prioritised

**Recommendation 7: understand the values and processes that underpin collaborative care.** Collaboration, even within teams^38^ and between sectors, should be prioritised in order to provide holistic, efficient and personalised care.^39^ Healthcare trust processes may differ geographically based on local processes and pathways for collaborative care. Knowledge of these pathways and processes may be key to identifying effective practices and where barriers lie; this could include a national study where service evaluations and interviews are undertaken to understand the values and processes of collaboration of learning disability MDTs with specialists and mainstream services. Furthermore, collaboration with learning disability MDTs would bring valuable contributions for recommendations 1–6.

### Strengths and limitations

Exploring the learning disability MDT perspective has provided rich insights from those who are directly involved in navigating mainstream healthcare services for their patients. For example, speech and language therapists were able to explain how communication difficulties affect practice in more detail; improvements were then linked to enhancing the use of health passports to facilitate safe care. This study has provided information that is valuable for healthcare professionals in various healthcare settings, such as primary care, or for emergency care practitioners who are frequently involved in the care of people with intellectual disabilities. Furthermore, from a service development perspective, particularly for mainstream services, there are significant barriers that need to be addressed.

Limitations included the fact that some members of the MDT were not represented in this study, including pharmacists, psychologists and social workers. Furthermore, this study presents the views of one healthcare trust, and the results may not be transferable to other MDTs.

In conclusion, this study has validated the realities and challenges faced by a learning disability MDT and their patients, while also providing empirical evidence for what is anecdotally known. Data are required before changes to practice, policy and services can occur to improve care provision for people with intellectual disabilities; the next steps may therefore include understanding the perspectives of other MDTs in the UK. A larger sample size, or a focus on the experiences of each discipline of those involved in the learning disability MDT, may allow for future studies to identify and compare themes.

## Data Availability

The data that support the findings of this study are available to other researchers on reasonable request from the corresponding author, M.M. The data are not publicly available, due to restrictions and the possibility of research participants being identified and their privacy compromised.
